# A qualitative exploration of professionals’ perspectives on the implementation of reablement intervention programs in community care

**DOI:** 10.1038/s41598-024-62047-6

**Published:** 2024-05-18

**Authors:** Ines Mouchaers, Lise E. Buma, Hilde Verbeek, Sandra Zwakhalen, Jolanda C. M. van Haastregt, Ellen Vlaeyen, Geert Goderis, Silke F. Metzelthin

**Affiliations:** 1 Department of Health Services Research, Faculty of Health Medicine and Life Sciences, CAPHRI Care and Public Health Research Institute, Maastricht University, P.O. Box 616, 6200 MD Maastricht, The Netherlands; 2 Living Lab of Ageing and Long Term Care, Maastricht, The Netherlands; 3 Department of Public Health and Primary Care, Academic Centre for General Practice, KU Leuven, Leuven, Belgium; 4 Cicero Zorggroep, Brunssum, The Netherlands; 5 Department of Public Health and Primary Care, Academic Centre for Nursing and Midwifery, KU Leuven, Leuven, Belgium; 6 Faculty of Medicine and Life Sciences, Hasselt University, Hasselt, Belgium

**Keywords:** Geriatrics, Public health, Rehabilitation

## Abstract

Reablement is considered a complex intervention due to its multicomponent, person-centered, holistic approach promoting older adults’ active participation in daily activities. It is important to consider the unique context in which complex interventions are implemented, as contextual factors may interact and influence implementation outcomes. As part of the European TRANS-SENIOR project, this qualitative study aimed to gain insight into professionals’ experiences with reablement implementation in Dutch community care. Using the Consolidated Framework for Implementation Research, four focus groups were conducted comprising 32 professionals. Two groups were formed: one at operational level, including therapists, nursing staff, social workers, and domestic support; and one at organizational/strategic level, including project leaders, managers, directors, municipality representatives and health insurers. Participating care organizations had at least 6 months of experience with deploying and implementing reablement. Findings reflected three themes: (1) strength of interdisciplinary collaboration; highlighting significance of sharing goals and beliefs, (2) integrating the reablement philosophy into the organization; underscoring managements role in fostering support across all organizational layers, and (3) achieving a culture change in the healthcare system; emphasizing current funding models impeding value-based care tailored to the individual’s goals and needs. The results offer valuable insights for implementation of complex interventions, like reablement.

## Introduction

Many countries stimulate aging in place, promoting older individuals to remain living at home independently for as long as possible^1–3^. Aging in place provides a stable foundation during times of significant change in the lives of older adults, promoting not only autonomy but also contributing to the preservation of their own identity^4,5^. It refers to the ability of older adults to live independently and comfortably their own homes or communities as they grow older. The concept encompasses not only the physical residence but also the community and social networks they are a part of Refs.^6,7^. Moreover, aging in place is often the preferred goal of older adults, despite increasing care needs^2,6,8,9^. Therefore, there is a need for sustainable, cost-effective, and patient-centered initiatives, focusing on improving quality of life and preventing or postponing institutionalization and in-patient care^10^. Reablement is considered an appropriate response to these needs^10^. It is a person-centered, holistic approach that promotes older adults’ active participation in daily life through social, leisure, and physical activities chosen by the older person in line with their preferences, either at home or in the community^11^. Reablement has some core principles and common features, for example, goal setting, an interdisciplinary approach, and a practice-oriented staff training^12–14^. A reablement trajectory is often time-limited and consist of several phases (i.e. initiation, intake, care plan, care delivery, and evaluation)^12^. Instead of taking over tasks, care professionals identify the capabilities and opportunities of individuals to maximize their independence by supporting them to achieve their goals, through training in daily activities, home modifications, assistive devices, and involvement of their social network^11,15–17^. Reablement is not a ‘one size fits all’-approach, meaning it is tailored to both the patient (i.e. their needs, preferences, and capabilities) and their environment^13,18^.

As the aging population continues to grow and individuals continue to live longer, the complexity of care needs and health issues also increases, often involving multiple health conditions^19^. To continue to meet these changing needs and adhere to the wish of older adults to age in place, care provision and health care interventions also become more complex. Reablement can be considered a complex intervention, which is typically difficult to implement in everyday practice^20^. Complex interventions generally include many interrelated components and factors and are provided and evaluated at different levels^20,21^. The complexity is more than the sum of all components, as other factors, for example, the implementation process, context, and participants, also have a major influence on achieving desired outcomes^21–23^. Lots of research has been done to unravel the barriers and facilitators influencing the implementation of complex interventions in health care (e.g., availability of resources, communication, culture, motivation and knowledge, etc.)^24–28^. Previous research has revealed important aspects related to the implementation of reablement, such as engagement of all parties involved, flexibility and professional autonomy, and shared vision and commitment^18,29–32^. However, some of these results were mainly based on researchers’ responses^29,31^, drawn from multilevel analyses^30^, or only based on the experiences of care staff^18,32^. Therefore, this needs to be further explored, especially from the perspective of multiple professionals involved in the implementation of reablement, since this has not been investigated previously. Moreover, it cannot be assumed that these factors are also applicable to the implementation of all reablement programs, across all settings. As complex interventions, like reablement services, are context-dependent^30,33,34^, it is important to consider the unique context in which they are implemented, as contextual factors such as organizational culture, networks and communication, and resources, may interact and influence implementation outcomes^35^. Therefore although reablement has been successfully implemented into usual care in, for example, Denmark and Australia^14^, it cannot be assumed that this applies to every context.

This study aims to gain insight into the experiences of healthcare professionals, management, and funders with the implementation of reablement in Dutch community care. By understanding and advancing reablement implementation, healthcare providers and policymakers are better equipped to successfully implement reablement both nationally and internationally. This study aims to address the following research question: how do professionals (i.e. operational, strategic, and organizational) experience the implementation of reablement in community care?

## Methods

### Design

The current study used a qualitative descriptive research design, in order to closely align interpretation and data analysis with participants’ responses. The study was guided by the Consolidated Framework for Implementation Research (CFIR), i.e. preparation interview guide and data analysis^36^. The CFIR is a meta-theoretical framework consolidating nineteen foregoing implementation theories. The framework can be used to prepare for innovation implementation and/or evaluative purposes to better understand factors influencing implementation outcomes, making CFIR both dynamic and valuable^37^. Moreover, the framework provides useful tools and aids to guide data collection, analysis, and reporting^38^. The Consolidated Criteria for Reporting Qualitative Studies (COREQ) checklist was used to strengthen the reporting of this study (Supplementary Appendix 1)^39^.

### Setting and participants

The study was conducted at three large care organizations that can be considered early adopters of reablement in the Netherlands (i.e. started the implementation of a reablement program at least six months prior to the start of the study). All organizations provide a range of services: from home care and (medical) treatment, to clinical rehabilitation and in-patient long-term care.

Criterion sampling was used to select professionals^40^. Eligible professionals had to be involved during the development, deployment, and/or implementation of reablement ensuring a well-rounded representation of professionals (i.e. variety of disciplines on operational, strategic, and organizational levels). Two groups of professionals were formed: (1) at the operational level, from here on referred to as care professionals, including occupational therapists, nursing care staff, physiotherapists, social workers, and domestic support workers, and (2) at the organizational or strategic level, from here on referred to as management, including project leaders, managers, directors, and policymakers, as well as, representatives from the municipality and health insurance companies, who played essential roles in the program’s implementation. Care organizations were contacted via email, stating the study’s background, objectives, and participation information. The project leaders within each organization were responsible for distributing the invitation to eligible professionals. Eligible participants received study details, including an information letter and informed consent form. Participants were requested to provide their written informed consent at the beginning of each interview.

### Data collection

Participant demographics (i.e., age, sex, and educational level, organization of employment, occupation, years of experience in the field, and years of experience with reablement) were collected through a questionnaire.

At each care organization, an on-site focus group was conducted with care professionals. Additionally, one overall online focus group was conducted with management. The separation of care professionals and management was maintained to create a safe environment when sharing their experiences. All focus group interviews were planned between December 2022 and February 2023 for a duration of one and a half or 2 h. No repeat interviews were conducted. All researchers conducting interviews were female and had prior experience with conducting interviews. Authors IM or LEB (Doctoral students) led the interviews and were assisted by one observer IM, LEB, or SFM (Assistant Professor). Interviews were guided using a semi-structured interview guide (Supplementary Appendix 2) based on the five domains of CFIR^36^, namely Intervention Characteristics, Outer Setting, Inner Setting, Characteristics of Individuals, and Process. The interview guide started with an open question about experiences with the implementation of reablement and what hindered or facilitated them herein. This first question was answered using sticky notes on which participants could write down hindering and facilitating factors. Subsequently, the sticky notes were clustered into themes and were discussed with the group. Follow-up questions were based on the five CFIR domains^36^ to obtain participants’ views on each domain. Field notes were taken during and after each interview and all interviews were audio-recorded to capture the intricate and nuanced data that characterize this type of research.

### Data analysis

Descriptive analyses of the background characteristics were performed using IBM SPSS Statistics (Version 25). Qualitative data was coded and analyzed using the qualitative data analysis software ATLAS.ti Windows (Version 23.0.8). All interviews were pseudo-anonymized and transcribed verbatim. For exploration and refining purposes, the data was first coded using inductive content analysis, the initial themes and categories were developed through iterative coding and discussions by IM, LEB, and SFM. Afterward, the data was analyzed and structured according to the CFIR domains using deductive content analysis^41^ with guidance from the CFIR information site^42^ while following the steps of the Framework Method as described by Gale et al.^43^. IM and LEB conducted the analysis collaboratively. The authors familiarized themselves with the data by reading the transcripts and taking notes. All coding was done independently, reviewed and compared, and discrepancies were discussed and resolved. The deductive coding was supplemented with the prior inductive coding for comprehensive analysis, ensuring no data was missed. Summarized data were organized into a matrix using Microsoft Excel 2016 (Microsoft Corporation, Redmond, WA, USA). This was reviewed and adapted by authors IM, LEB, and SFM.

### Rigor and reflexivity

Multiple strategies were used to increase rigor in terms of credibility, dependability, and conformability^44^. Member checking was done during and at the end of each focus group using interpretation checks, and afterwards with summaries of key findings providing participants with the opportunity to respond, which was used by one participant. Investigator triangulation was applied in both data collection and data analysis. The iterative process allowed for re-examining initial findings using insights that emerged during analysis. Results were discussed within the research team until consensus was reached. To reflect on the process, choices made and intermediate results a research diary was used by IM and LEB. During data collection, objectivity was ensured by consciously formulating the posed questions and prompts. However, knowledge of the subject matter and close involvement in practice may have influenced the decisions during data analysis and thematic selections. These decisions were discussed within the research team on a regular basis to prevent such influences, involving members less directly involved in practice.

### Ethics

The study was conducted according to the guidelines of the Declaration of Helsinki, and approved by the Ethics Committee of Maastricht University, Faculty of Health, Medicine and Life Sciences (approval number FHML-REC/2022/126). Participants voluntarily signed informed consent after they were fully informed about the purpose and procedures of the study and had the opportunity to ask additional questions or raise any concerns. The written informed consent stated that participation was completely voluntary and withdrawal from the study was possible at any moment, without providing a reason, by contacting one of the researchers (IM/LEB/SFM).

## Results

In total, 32 professionals participated in the study. Eighteen professionals were involved in the three focus group interviews with care professionals. The care professionals included: occupational therapists (n = 4), physiotherapists (n = 4), district nurses (n = 3), certified nursing assistants (n = 2), a nurse practitioner (n = 1), elderly care physician (n = 1), community consultant (n = 1), consultant informal care (n = 1), and a work planner domestic support services (n = 1). Twelve professionals were involved in the online focus group with management. Management included: directors (n = 3), managers (n = 3), project leaders (n = 3), an implementation coach (n = 1), a policymaker from the municipality (n = 1), and a team leader (n = 1). Additionally, two healthcare insurer representatives participated in the online focus group at organizational and strategic levels, from here on referred to as funders. Table 1 provides an overview of all participants involved, including information about their age, sex, educational level, discipline or professional role within the organization, years of experience within their discipline and reablement.

**Table 1:** Background information of participants (n = 32).

	Care professionals (n = 18)	Management (n = 12)	Funder (n = 2)
Age (years), mean (SD)	41.7 (11.0)	48.1 (9.6)	37 (0.0)
Sex, n (%)			
Male	2 (11.1)	2 (16.7)	1 (50.0)
Female	16 (88.9)	10 (83.3)	1 (50.0)
Educational level*, n (%)			
Intermediate	3 (16.7)		
High	15 (83.3)	12 (100.0)	2 (100.0)
Organization, n (%)			
Care organization A	8 (44.4)	3 (25.0)	
Care organization B	5 (27.8)	5 (41.7)	
Care organization C	5 (27.8)	3 (25.0)	
Municipality		1 (8.3)	
Healthcare insurer			2 (100.0)
Years of experience, mean (SD)			
Professional role	10.3 (8.0)	7.5 (8.9)	3.5 (0.7)
Reablement	1 (0)	1.6 (0.7)	2.0 (1.4)

*Intermediate: Intermediate vocational or higher secondary education; High: Higher vocational education, university.

The results reflected three overarching themes: (1) strength of interdisciplinary collaboration, (2) integrating the reablement philosophy into the organization, and (3) achieving a culture change in the healthcare system. The data corresponding to the domains and constructs of the CFIR are presented as “*(Domain: Construct)*”.

### Strength of interdisciplinary collaboration

This theme describes how aspects related to the architecture and application of the programs impacted implementation. However, the key focus was on collaboration, both internal and external, and was mainly related to the CFIR domains: Inner setting, Outer setting, and Intervention Characteristics.

#### Internal collaboration

All care professionals perceived reablement’s interdisciplinary character as facilitating (*Inner Setting: Networks & Communications*). They mentioned a more intensive collaboration due to increased insight into each other’s profession and capabilities, which was also noticeable beyond the program. In addition, care professionals indicated that, together with the client, they gave more consideration to which professionals should be involved. In their view, the structured team meetings improved communication, and the shared set of goals created shared ownership. These facilitating factors were endorsed by management.

That [collaboration] really has improved. You also know what everyone does, what you can find each other for. […] It’s as if the threshold has somehow disappeared. They know who you are, they know what you do and they also come to you with different questions about very different things, not just reablement*.* (Occupational therapist, Care organization C)

However, most care professionals also indicated hindrances, such as scheduling meetings and intake assessments, limited access to others’ reports, and lack of overview of the care professionals involved (*Inner Setting: Structural Characteristics*). Furthermore, management indicated unclear task distribution among professionals with coordinating roles sometimes caused tension. For example, when the occupational therapist took on a coordinating role, this sometimes felt threatening to district nurses or case managers.

But, where the friction often arises is in the coordinating role [...] that has nothing to do with professionals feeling more or less than another. But, that they […] don’t quite understand what their [...] role looks like within that reablement program, and that the coordination might temporarily lie with the occupational therapist […], or temporarily with the district nurse. If those agreements are unclear, that’s the feeling you get. (Director, Care organization A)

#### External collaboration

Participants indicated a lack of structural collaboration with external professionals, including domestic support workers, general practitioners, case managers, and municipalities (*Outer Setting: Cosmopolitanism*). Especially the lack of collaboration and involvement with general practitioners was experienced as hindering due to lack of background information and was reported to hinder clients’ independence. This was also the case when external care professionals were involved who did not follow reablement principles.

We also get regular referrals of clients saying, ‘Go take a shower twice a week and pretend to be worse than you are, because then you might get a long-term care indication and then you can move [to a nursing home].’ Because there are care professionals [outside the organization] who think they should move. (Physiotherapist, Care organization B)

### Integrating the reablement philosophy into the organization

This theme reflects on the role management played in integrating the reablement philosophy throughout all actors involved. Their efforts to establish a strong foundation were considered crucial for successful implementation. Additionally, this theme reflects on what influenced the necessary readiness for change, both for professionals as well as clients and their informal caregivers. These findings were mainly related to the domains: Inner setting, Outer setting, Characteristics of the individual, and Process from the CFIR framework.

#### Role of management in program integration

Care professionals felt both facilitated and hindered by management; while they experienced freedom to experiment with reablement, they also expressed a need for clearer boundaries (*Inner Setting: Implementation Climate—Learning Climate*). Additionally, home care teams experienced change fatigue due to the simultaneous implementation of numerous projects during the time reablement was implemented (*Inner Setting: Implementation Climate—Relative Priority*). Most care professionals expressed that they felt unheard by management when raising issues and missed feedback and follow-up (*Inner Setting: Networks & Communications, Readiness for Implementation—Leadership Engagement*). They also mentioned a lack of clarity on the program’s purpose, which resulted in mismatched expectations of care professionals (*Inner Setting: Implementation Climate—Goals & Feedback*). Management endorsed the need for a communication strategy beyond just providing information. Lastly, care professionals felt pressure to deliver positive results due to high expectations from both management and researchers.

Policy, management, ministry and so on all come up with plans. We have to implement it, but there is no connection. We have to pass on signals all the time. It takes an awful lot of time. Moreover, it is very incomplete, because we have to put it into words, […] then you have to meet with your quality officer again. […] I just don’t have time for this. (Community consultant, Care organization C)

However, participants also emphasized the vital role that management played to sustain the reablement philosophy within their organizations and acknowledged management’s successful efforts. For example, hiring an implementation coach, conducting regular evaluations and project group meetings (*Process: Planning, Engaging, Reflecting & Evaluating*), sharing success stories, and establishing low-threshold communication with care professionals and clients (*Process: Engaging*), were mentioned as facilitators for the implementation of reablement to resonate both inside and outside the organization (*Inner Setting: Readiness for Implementation, Leadership Engagement*).

What has also helped us a lot is the success stories […] that are there, and to celebrate and share them. And collaboration […] also very beneficial. Because then they will have achieved something together which they can be proud of. And well that totally contributes to the whole process of getting […] the change going, and to be especially mindful of that. (Manager, Care organization B)

#### Readiness for change

Nearly all participants indicated that the implementation of reablement programs led to a change in perspective among care professionals, facilitating interdisciplinary collaboration and promoting equality and sustainability. (*Characteristics of the Individual: Knowledge & Beliefs about the Innovation, Individual Stage of Change*). However, a lack of mutual beliefs (e.g. external professionals) was perceived as hindering (*Outer Setting: Cosmopolitanism, Characteristics of the Individual: Knowledge & Beliefs about the Innovation*). Care professionals’ readiness for change was said to be influenced by personal factors such as years of experience, educational level, and motivation (*Characteristics of the Individual: Individual Stage of Change, Other Personal Attributes*).

You notice that the people who were already working in home care […] find it much more difficult [to change]. Because then it’s like, ‘Oh, I’ll just do that quickly and then I’ll finish earlier and I can move on to the next one quicker, so no one has to wait’. But with the younger generation, you notice that it really is easier [to change]. (District nurse, Care organization A)

Care professionals indicated that mainly personal factors among clients and informal caregivers influenced their readiness for change. For example, their motivation, expectations, whether they had been receiving care for a long time, perceptions of care among the older generation, knowledge, and health literacy (*Characteristics of the Individual: Individual Stage of Change, Other Personal Attributes*)*.* To facilitate change, and consequently the success of the program, care professionals stressed the importance of conducting the intake and goal-setting in a way that helps clients and informal caregivers become aware of the necessary steps to achieve their goals and creates a sense of ownership (*Characteristics of the Individual: Knowledge & Beliefs about the Innovation*).

We really ‘do with’ and most people are really still ‘doing for’ […] that does clash regularly. Clients also feel, and I think this is especially true for wealthier people, that they are entitled to a lot of things. Because they have worked hard all their lives and paid a lot and now we will have to [care for them]. […] I am often told, ‘Yes, you could just come anyway, because we have been paying health insurance all our lives, so we are entitled to this, so you should do it.’ (District nurse, Care organization A)

### Achieving a culture change in the healthcare system

This theme reflects on the shift towards a ‘doing with’ approach rather than ‘doing for’ approach, which means that instead of taking over tasks from clients, self-management is stimulated. This empowers clients to actively participate and take ownership, with a focus on prevention, which matches the ongoing care transformation in the Netherlands. It also explores the societal responsibility that healthcare organizations bear in this transformation. Participants discussed funding issues and the prerequisites for successfully navigating this transition. These findings were related to the domains: Inner setting, Outer setting, Characteristics of Individuals, and Intervention Characteristics from the CFIR framework.

#### Funding

All participants perceived current funding of reablement as hindering to the deployment of the reablement programs as desired. Current insurance reimbursement in the Netherlands falls short for some aspects of reablement, for example, team meetings, physiotherapy, and a sufficient amount of occupational therapy (*Outer Setting: Needs & Resources of Those Served by the Organization*). Subsequently, care professionals mentioned that clients were reluctant to pay for additional non-reimbursed costs and therefore possibly would not participate in the program (*Inner Setting: Readiness for Implementation—Available Resources*). Moreover, management indicated that the possibility of implementing reablement more preventatively is also hindered by the financial and administrative rules of the current reimbursement system in primary care. Both management and care professionals therefore expressed a need for a form of funding that is not project-based and facilitates integral reimbursement (*Outer Setting: Needs & Resources of Those Served by the Organization*).

We also hope that eventually there will be an integral reimbursement for this issue so that you can really look specifically at the client: ‘Hey, what do they need now?’ And that you don’t have to weigh up every time, like: ‘They can get a bit more occupational therapy [reimbursed] now, so we use that a little bit more, because physiotherapy is not in the [reimbursement] package.’ You don’t want to look at it that way. You really want to look at: ‘Hey, what are the goals and […] what can we deliver to reach that?’ (Project leader, Care organization C)

From management’s perspective, the current project-based approach to implement and fund reablement is hindering its permanent positioning within the healthcare system (*Inner Setting: Implementation Climate—Relative Priority*). They felt this approach leads to perceptions that reablement is merely an add-on, lacks commitment, and may not replace or supplement existing care services effectively. One of the funders endorsed that not having integral funding is hindering, but emphasized that they require to know what the added benefits of reablement are compared to usual care (*Intervention Characteristics: Trialability*).

We are really not looking to know it all. We don’t need huge thick files to back it up, but we do want to be able to compare it. […] What is the difference with usual care, except, that clients are more in the lead and have more autonomy. I’m all in favor of that, but can we also make it clear what it means? What the other way of working entails, compared to the old way? (Funder, Care organization C)

#### Care transformation and prerequisites

Management mentioned that they felt external pressure due to the societal mission set by the Dutch government, which emphasizes the need for affordable and accessible healthcare (*Outer Setting: Peer Pressure, External Policy & Incentives*). Care professionals and management consider reablement an essential change to address the growing demand for care (*Inner Setting: Tension for change*). They see it as a way to offer more preventative care, reducing clients’ dependency on services, and possibly delaying more complex care (*Inner Setting: Implementation Climate—Relative Priority*).

What I sincerely believe is that reablement will very much contribute on prevention. That this will ultimately keep people out of long-term care facilities, or at least not until a later stage. We also see now—certainly the group that is currently applying through the municipality—we see that when on time... Being involved much earlier, that’s really going to result to needing less hospital care and other expensive forms of care. (Project leader, Care organization C)

To implement reablement on a larger scale, management believes that maintaining a dialogue with professionals and expanding collaboration with other organizations is crucial (*Inner Setting: Implementation Climate—Relative Priority*). However, they also mentioned that the time and effort required to establish behavioral change among care professionals may be hindering. They also felt this transformation was insufficiently supported by national policies. In their view, the current lack of prerequisites, laws, and regulations needed to implement reablement in the Dutch context are largely absent (*Outer Setting: External Policy & Incentives*). Additionally, management believes that, besides staff shortages, they have a responsibility to facilitate a new professional standard, as current standards are lacking and missing the necessary skillset needed for effective reablement delivery (*Characteristics of Individuals: Individual Stage of Change*).

We need to move towards a new professional standard, especially for district nursing. And you don’t achieve that by quickly scaling up. I personally believe that in the long run, once you have it implemented correctly, you can enable many clients to take care of themselves in the community with district nursing, reablement, and potentially other aids. [...] The entire program must be delivered by occupational therapists. And we don’t have 10,000 of them either. So […] I think we shouldn’t think it [upscaling] is just done like that. Because, in my view […] it’s moving too fast. It’s too complicated for that. (Manager, Care organization B)

## Discussion

This study aimed to gain insight into professionals’ experiences with the implementation of reablement, a complex interdisciplinary intervention in Dutch community care. The findings reflected three overarching themes: (1) strength of interdisciplinary collaboration, (2) integrating the reablement philosophy into the organization, and (3) achieving a culture change in the healthcare system. Through the perspective of multiple professionals on different levels (i.e. operational, strategic, and organizational), the findings reflected the characteristic interrelations of different components and influences associated with the implementation of complex interventions.

Care professionals experienced improved interdisciplinary collaboration, enhanced understanding of each other’s roles, and shared ownership, which was mainly facilitated by structured team meetings and shared goals. Interdisciplinary collaboration is experienced as positive and essential amongst healthcare professionals working with reablement^45–47^, for example, in getting perspectives from different angles^48,49^. However, other studies endorsed the hindering factors (i.e. scheduling conflicts and lack of accessible reports) found in our study^47,50^. The most important finding relates to the challenges encountered due to a lack of mutual beliefs, structural collaboration and alignment with external parties, and consequently, the extent to which all involved care professionals adhered to the reablement principles. Therefore, causing ambiguity and possibly leading to suboptimal contributions of some team members^45^. However, competing logics among involved parties should not hinder implementation^51^. This can be strengthened when all parties work towards a shared goal, align their beliefs, and establish more structured forms of collaboration^52^. In addition, it is essential to enable care professionals to learn from each other’s perspectives thereby complementing and enhancing their skills^49^.

The success of the implementation seems to depend on the integration and upkeep of the reablement philosophy throughout all professionals involved. In accordance with prior research^49^, the most important finding was management’s pivotal role in sustaining the reablement philosophy within the organizations. Especially in these contexts, where the collective shift of mindset and professional role identity depends on the expectations of multiple professionals, achieving cultural change relies on rethinking institutional logics (i.e. shared beliefs and values determining behavior, shaping actions and decisions) and interrelationships^52^. Management’s initiatives were seen as facilitating the implementation and have proven to be effective when applied within all layers of the organization^47,53^. However, Fakha et al.^27^ confirm the lack of continuity indicated by the participants (i.e. disrupted information flow, communication, etc.), can impede the implementation of innovations. Establishing strong external networks and clear communication are essential to foster implementation^27^. Therefore, it is recommended to maintain open communication across all organizational layers and provide time, space, and resources necessary to reconsider institutional logics^49,52,54^. This engagement can be further enhanced when staff is given the opportunity to provide input and feedback (e.g. during interactive sessions with management), through which they can voice their opinions and concerns, ask questions, offer suggestions, and feel heard^49,54,55^.

It became evident that funding and supporting regulations in the Netherlands were perceived as hindering for nationwide implementation of reablement. Current funding and reimbursement schemes fall short of covering all costs related to reablement programs and their accompanying interdisciplinary collaboration^56^. Consequently, this hinders care professionals from delivering care based on the client’s goals and needs, as the care provided is dictated by reimbursement criteria. This is in line with Parsons et al.^34^, who emphasize the need for a funding model that facilitates goal-oriented, holistic, and person-centered home care. Both management and care professionals expressed a need for a more integrated form of funding as the current fee-for-service model does not encourage value-based care, fosters fragmented healthcare, and encourages volume-based incentives^14,57^. In addition, the current model does not incentivize preventive care and early interventions^57,58^. Moreover, a strong and shared national vision regarding a new way of delivering home care is needed (i.e. supporting organizational procedures and national policies)^17^. An integrated funding model could potentially provide a solution providing high-quality care tailored to the client’s needs, ultimately reducing healthcare costs by promoting preventive care and early interventions^59,60^.

Some methodological considerations have to be made. First, we used a criterion sample of professionals based on the personal judgment of the research team and previous collaboration with the professionals, which could lead to selection bias of more motivated participants. However, it allowed us to obtain insight from the professionals who were closest to the implementation process. Second, to minimize time investment and effort required from organizations and professionals, the decision was made to conduct four focus groups instead of pursuing data saturation. In addition, the uneven distribution of participants raised a concern, especially since one of the interviews involved 14 participants, potentially limiting the representation of some participants in our combined analysis. Nevertheless, our study presents a methodologically sound and comprehensive understanding of the factors influencing a nationwide implementation of reablement from an implementation science perspective, for example, by using a well-known framework (i.e. CFIR^36^) to guide our study. It is important to note that the CFIR framework was updated during the preparation of this research^61^. The revised version highlights the importance of including the end-users’ perspective which ensures care meets their needs, enhancing person-centered and effective healthcare^62^. As a consequence, our study only reflects clients’ experiences through professionals’ views.

Notwithstanding these limitations, this study offers valuable insights for the implementation of reablement across diverse (international) settings and offers lessons applicable when implementing complex interventions. It can serve as a starting point to determine suitable, and effective strategies to address the identified influences on implementation. Linking our findings to CFIR provides sufficient guidance to choose appropriate strategies for implementation^63^. Future research could quantify a mix of key influences and explore their impact due to reablement’s context-specific character, further tailoring the chosen strategies. For practical application, care organizations should foster an innovation climate promoting open communication throughout all layers of the organization, as well as with service users. Policy should prioritize adopting an integrated funding model, which offers structure when implementing complex, interdisciplinary, interventions such as reablement; especially early on in the care process.

### Supplementary Information

Supplementary Information 1.

Supplementary Information 2.

## Data Availability

The data presented in this study are available on request from the corresponding author. The data are not publicly available due to their containing information that could compromise the privacy or consent of research participants.
