# Supplementary material for: A qualitative exploration of professionals’ perspectives on the implementation of reablement intervention programs in community care
Source: Sci Rep. 2024 May 18;14:11391. doi: 10.1038/s41598-024-62047-6 (PMC11102453; doi:10.1038/s41598-024-62047-6)
Supplement: Supplementary file 2 — Supplementary Information 2. [file 41598_2024_62047_MOESM2_ESM.pdf]

<u>Appendix 2 – Semi-structured interview guide</u>

A. Overall experiences with implementing reablement

1. Which factors did you experience as facilitating when implementing reablement?
    a. Why was this a facilitating factor?
2. Which factors did you experience as hindering when implementing reablement?
    a. Why was this a hindering factor?

B. Domain 1: Intervention characteristics

3. What challenges did you face during the implementation of reablement?
4. How can these problems be solved?
5. What do you think are the advantages and disadvantages of reablement compared to conventional home care?

C. Domain 2: Outer setting

6. To what extend has current legislation and regulations influenced the implementation of reablement?
7. To what extend have other factors outside the organization influenced the implementation of reablement?

D. Domain 3: Inner setting

8. To what extend has the organization facilitated or hindered the implementation of reablement?
9. In what way has communication within the organization and within the reablement team influenced the implementation of reablement?
10. To what extend does reablement fit within the organization's current policy and how did this affect the implementation of reablement?

E. Domain 4: Characteristics of the individual

11. Which people supported you in the implementation of reablement?
    a. How did they support you?
12. Which people hindered you in the implementation of reablement?
    a. How did they hinder you?

F. Domain 5: Process of implementation

13. Was the reablement program implemented as intended?
    a. If not, why not?
14. What strategies were used to implement the reablement program as planned?
